# Post‐Trabeculectomy Refractory Shallow Anterior Chamber: A Case Highlighting Overlooked Patient Behavior

**DOI:** 10.1002/ccr3.70970

**Published:** 2025-09-25

**Authors:** Md. Iftekher Iqbal, Fariah Osman

**Affiliations:** ^1^ Glaucoma Department Ispahani Islamia Eye Institute and Hospital Dhaka Bangladesh; ^2^ Ispahani Islamia Eye Institute and Hospital Dhaka Bangladesh

## Abstract

A shallow anterior chamber (AC) after glaucoma filtration surgery is a common early postoperative complication that can be vision‐threatening. So, early recognition of the causative factors and prompt management are necessary to avoid such devastating complications after surgery. This case report presents a 33‐year‐old patient with primary angle closure glaucoma who initially presented with an acute angle closure crisis in both eyes. Despite having maximum tolerated anti‐glaucoma medications, his target IOP was not achieved, and he later underwent augmented trabeculectomy with mitomycin C in his right eye. However, frequent swallowing of the AC was noticed in the early postoperative days. Revision surgeries with AC reformation were done after initially dealing with the unrecognized patient factor and maintaining good vision with well‐controlled intraocular pressure and a well‐formed AC. After incisional glaucoma surgeries, it is essential to warn patients about external factors contributing to postoperative complications, like shallow AC.

**Summary**

- Other than surgical factors, patient‐related factors may also contribute to the post‐trabeculectomy shallow AC.

## 1 Introduction

When compared to other glaucoma surgical techniques, trabeculectomy is the most often done surgical operation in medically uncontrolled glaucomatous eyes and is currently regarded as the gold standard since it was first introduced by Cairns in 1967 [1, 2].

One of the major complications of trabeculectomy surgery is a shallow anterior chamber (AC), with or without hypotony. It may result from malignant glaucoma, overfiltration, leaky bleb, or serous choroidal detachment [3, 4]. Antimetabolite adjunctive therapy (mitomycin C: MMC, 5‐fluorouracil: 5‐FU) enhances postoperative aqueous outflow, significantly raising the risk of overfiltration, a frequent early postoperative complication [1].

Corneal endothelial decompensation, cataract development or progression, and synechiae development are examples of secondary problems that might result from a shallow or flat AC [5, 6].

This article details a case of primary angle closure glaucoma that was treated by trabeculectomy with MMC and other procedures to address the AC's refractory shallowing. We also draw attention to patient‐related factors that may contribute to the development of shallow AC after surgery, which have not received enough attention in the literature yet.

## 2 Case Presentation

### 2.1 History

A 33‐year‐old Bangladeshi male came with a history of bilateral ocular discomfort, blurry vision, redness, photophobia, and frequent headaches for a week. During this period, he also mentioned being nauseated sometimes but never having vomited. He didn't give any history of ocular trauma or any ocular or systemic medications for any of these, other than taking oral paracetamol a few times to relieve pain. But the symptoms persisted, and he later visited for an eye examination. He also mentioned occasional mild ocular pain, photophobia, redness, blurry vision, and colored halos for the last 3 to 4 months, but mostly in the left eye at that time. He did not have any systemic disorders and no significant family history, especially for glaucoma‐related blindness.

### 2.2 Clinical Examination and Diagnosis

During the ocular examination, his best‐corrected visual acuity with Snellen's acuity chart was 6/36 in the right eye (RE) and hand movement (HM) with a projection of rays (PR) in all four quadrants in the left eye (LE). Intraocular pressure (IOP) was raised in both eyes and measured with a Goldmann applanation tonometer at 35 and 40 mmHg in RE and LE, respectively. There was conjunctival and circumciliary congestion, moderate corneal edema, and extremely shallow AC depth (van‐Herick Grade 1) with apparently clear lenses in both eyes. The pupil was mid‐dilated, round, and regular in shape with a sluggish reaction in the RE, where the left pupil showed mid‐dilation with the presence of a relative afferent pupillary defect. As the media were hazy due to corneal edema in both eyes, gonioscopy, optic discs, and retinal evaluation were not appreciated during that time. Thus, he was clinically diagnosed as a case of primary angle closure glaucoma with acute angle closure crisis in both eyes.

### 2.3 Treatment

After proper counseling about the disease process and ensuring no contraindications to any of the glaucoma medications, we prescribed oral acetazolamide (500 mg) at that time, followed by 250 mg twice daily for another 3 days, along with potassium chloride (600 mg) in halves, twice daily for 3 days. A topical combination of timolol maleate (0.5%) and brimonidine tartrate (0.2%) twice daily and brinzolamide (1%) thrice daily, along with prednisolone acetate (1%) four times daily, was started. Then the follow‐up was scheduled after 7 days.

**FIGURE 1:**
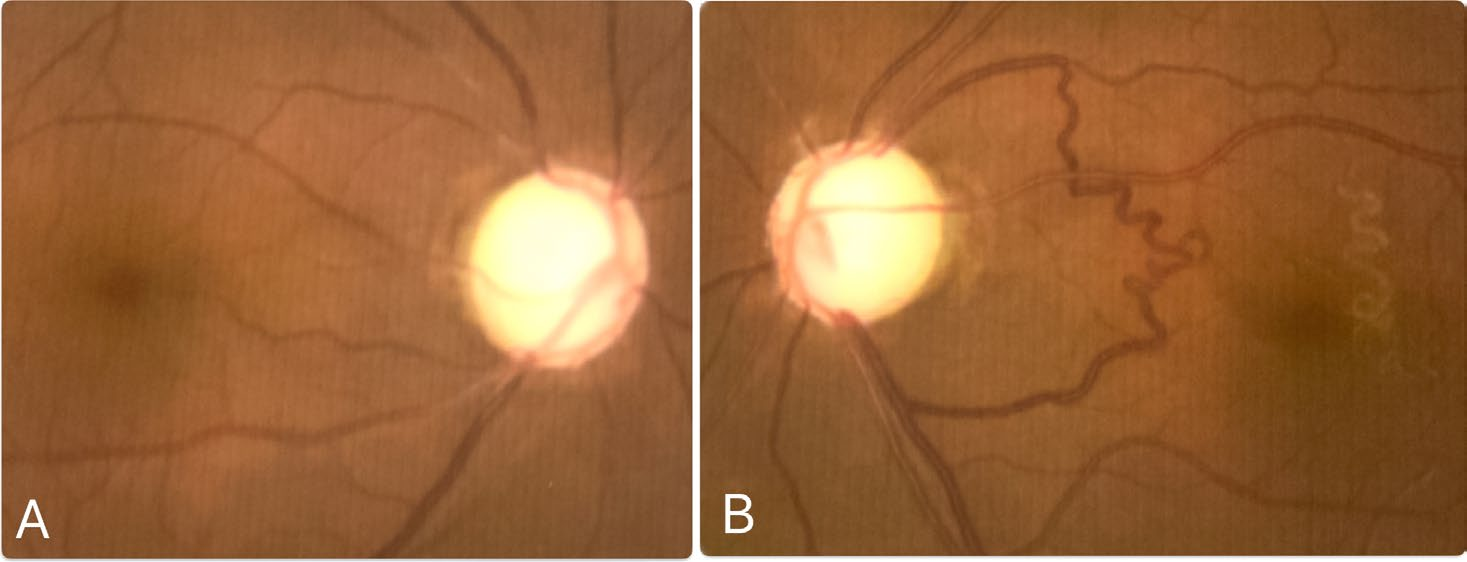
Color fundus image: (A) right eye, (B) left eye.

**FIGURE 2:**
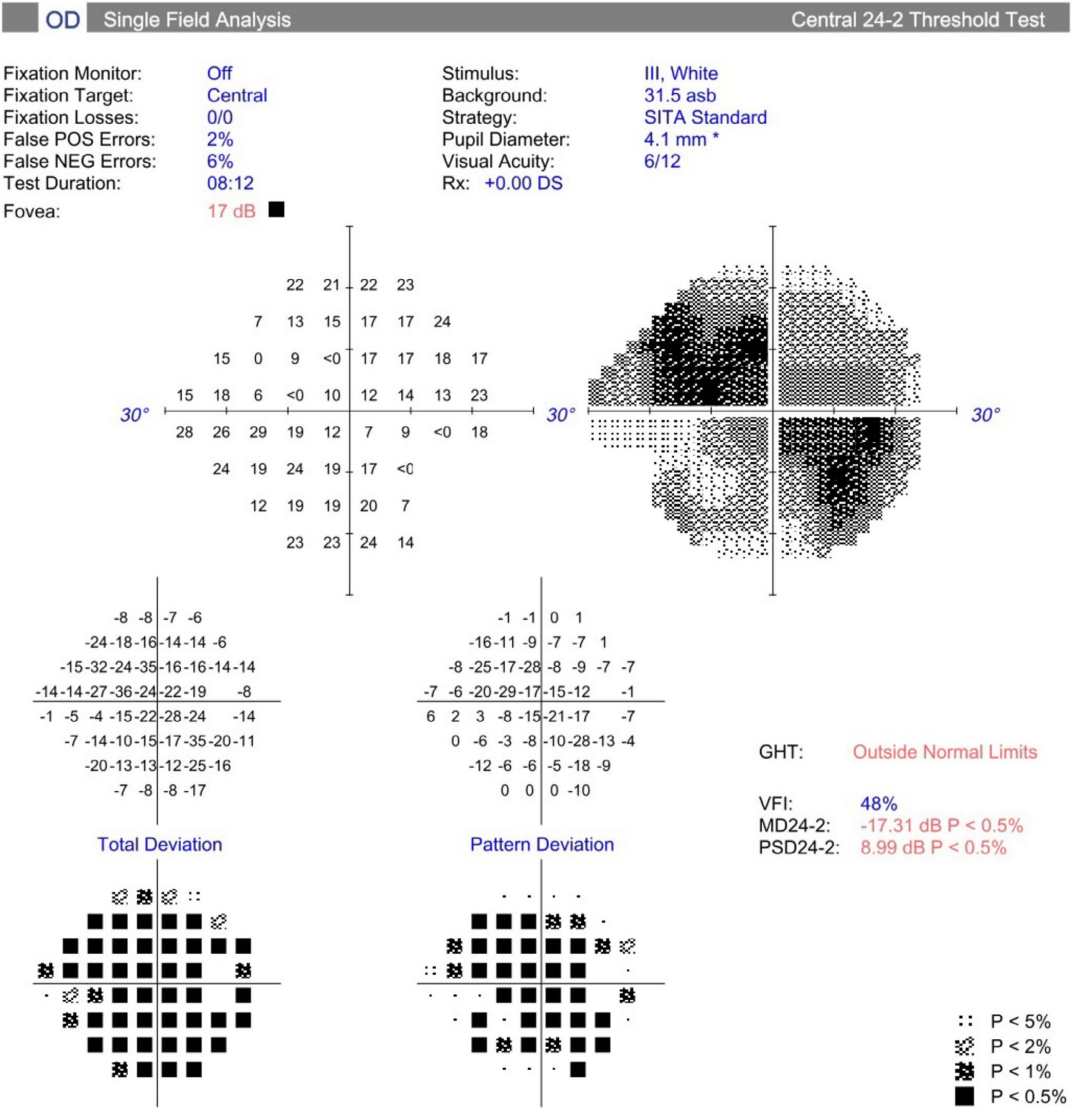
Humphrey visual field analysis (24–2‐central) of right eye.

During his follow‐up period, his BCVA was improved to 6/12 in the RE, but in the LE, remained HM with a PR. IOP with maximum tolerated anti‐glaucoma medications was 24 mmHg in RE and 30 mmHg in LE, with no corneal edema, grade‐0 in all four quadrants of the AC angle on gonioscopy (Shaffer's grading), and the cup‐to‐disc ratio of 0.80:1 in RE (Figure 1A), 1:1 in LE (Figure 1B) with no notable retinal pathologies and corresponding Humphrey visual field (HVF) changes in RE (Figure 2).

As the target IOP was not achieved, we planned augmented trabeculectomy with 0.02% mitomycin‐C (MMC) in both eyes (RE initially, then LE). Following the tenets of Helsinki, one of the glaucoma specialists (Iqbal M) performed the uneventful surgery in the RE. This procedure was done under surface anesthesia. Initially, subconjunctival 0.1 mL of 0.02% MMC was injected and left for around 60 s. Then fornix‐based conjunctival peritomy was done, and the MMC was thoroughly washed out. After securing the bleeding points with minimal cautery, a 4 × 4 mm triangular scleral flap was created. Then paracentesis with a 2.8 mm keratome, sclerostomy with Kelly's punch forceps, and surgical peripheral iridectomy were done sequentially. With air tamponade in the AC, the triangular scleral flap was closed with a 10–0 nylon suture at the apex. Then the AC was reformed with balanced salt solution (BSS), and after confirmation of no leakage, the conjunctiva was closed with an 8–0 polyglactin suture in a watertight manner. Finally, the surgery was finished by injecting 0.5 mL of intracameral preservative‐free moxifloxacin (0.8 mL) and keeping the eye patched for 2 h post‐operatively.

## 3 Conclusion and Results

After removal of the eye patch, the patient was advised to instill topical moxifloxacin (0.5%) 6 times daily for a month, prednisolone acetate (1%) hourly for a day, then tapered over a month, and homatropine (2%) twice a day for 7 days. He was also advised to take oral paracetamol if needed for pain.

**FIGURE 3:**
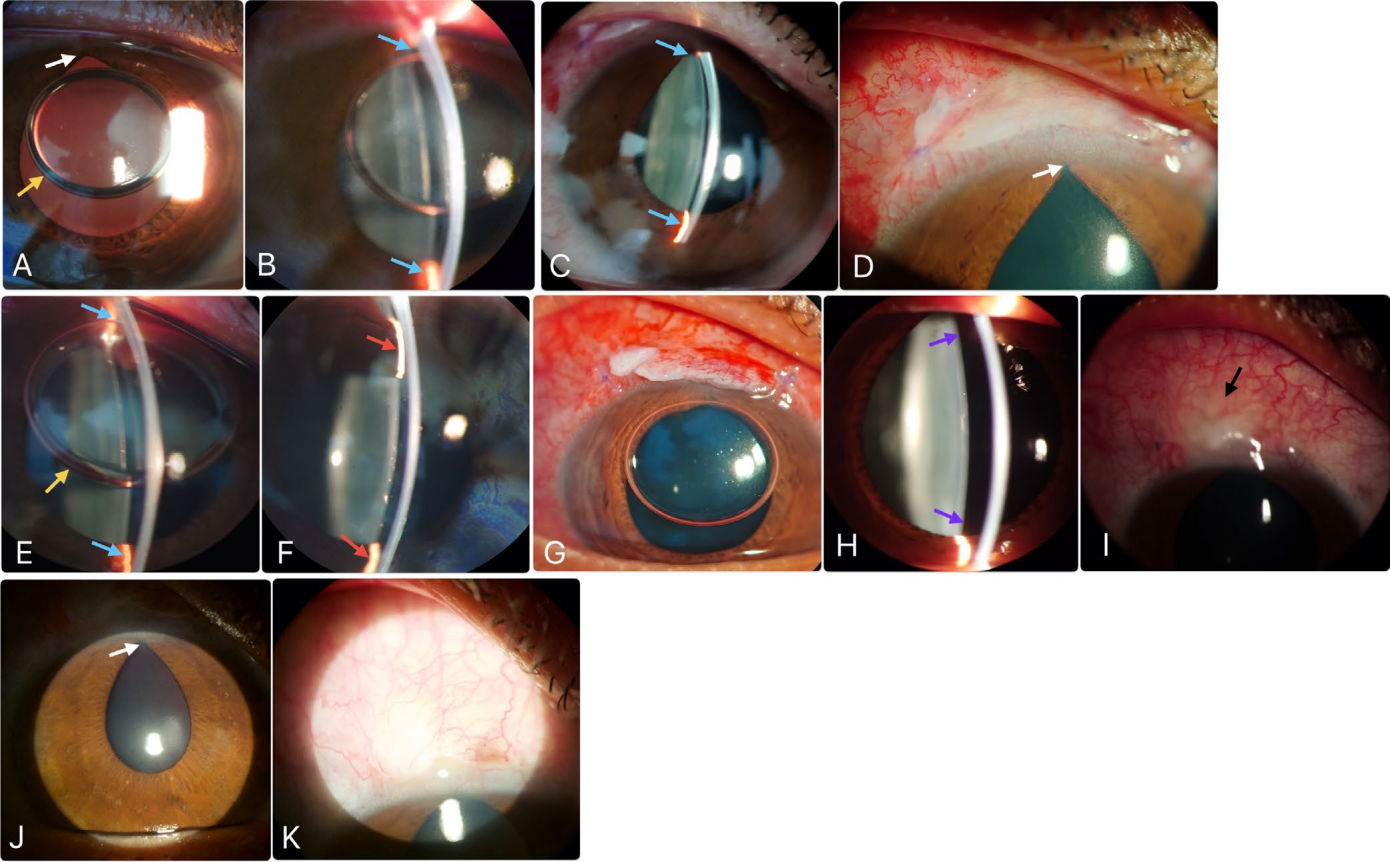
(A, B) First postoperative day status after trabeculectomy—(A) air in anterior chamber, dilated and peaked pupil (white arrow), (B) shallow anterior chamber with irregular depth (blue arrows); (C, D) Eight postoperative day status after trabeculectomy—(C) shallow anterior chamber (blue arrows), (D) peaked pupil; (E, F) First post‐revision status—(E) shallow anterior chamber (blue arrows) with air (yellow arrow) on day 1, (F) shallow peripheral anterior chamber (red arrows) on day 11; (G–I) Second post‐revision status—(G) well‐formed anterior chamber with air on day 1, (H) well‐formed, deep anterior chamber (purple arrows) in day 11, (I) highly vascular flat bleb (black arrow) on day 11; (J, K) Three months post‐trabeculectomy status—(J) corectopia (white arrow), (K) diffuse, moderately vascular, functioning bleb.

On the first postoperative day (POD), his BCVA was 6/60 with an IOP of 12 mmHg without any glaucoma medications. Slit‐lamp examination (Figure 3A,B) revealed a clear cornea but a shallow AC with irregular depth. There was air in the AC with 1+ cells and flare, and a pharmacologically dilated pupil. However, there was no bleb leakage (negative Seidel test) and no overfiltration with a flat bleb. As there was no lens‐corneal touch, we decided to observe the patient and advised him to come for follow‐up after 7 days.

On the 8th POD, the patient visited with an improved BCVA of 6/36 and an IOP of 16 mmHg. But the AC remained shallow (Figure 3C), with the peaked pupil superiorly (Figure 3D). Then we decided to do the revision surgery, and the primary surgeon did that by repositioning the iris from the sclerostomy site, putting two additional sutures with 10–0 nylon on either side of the triangular scleral flap, and reforming the AC with BSS and air.

On the 9th POD (1st POD after 1st revision), his BCVA was 6/36 with 20 mmHg IOP. But the AC was shallow (Figure 3E). At this point, he denied any eye rubbing, heavy work or lifting weights, constipation, and violent coughing or sneezing. As he was maintaining good vision and IOP, we followed up with the patient for another 10 days.

On the 19th POD (11th POD after 1st revision), he came with a shallow AC (Figure 3F), maintaining the same vision as before and an IOP of 18 mmHg. As he denied extraneous physical work or any violent coughing or sneezing from the beginning, we asked about his recent sexual activities. Finally, he told us about his recent marriage and admitted that he had sex every night after the procedures. This time, the revision surgery was done, ensuring a well‐formed AC, no leakage from wounds, and water‐tight conjunctival closure, and strictly advised for sexual abstinence for 2 weeks.

On the 20th POD (1st POD after 2nd revision), the patient came for follow‐up with BCVA 6/36 with IOP of 14 mmHg and a well‐formed AC with air in AC (Figure 3G). Later, he was advised to follow up after 10 days.

30th POD (11th POD after 2nd revision), the patient came with BCVA 6/12 with a well‐formed AC (Figure 3H). However, a highly vascular flat bleb (Figure 3I) led to a raised IOP of 24 mmHg. Along with the previous postoperative topical medications, we added timolol maleate (0.5%) twice daily and educated him to give mild digital pressure in the lower lid region with the eye closed and looking up to increase outflow and reform the functioning bleb. Since then, the patient has been on regular follow‐up, maintaining good vision with target IOP with a single antiglaucoma medication Table 1.

**TABLE 1:** Summary of Postoperative Course.

Postoperative day	Event	Findings	Key points	Outcome
Day 1	Initial follow‐up	Shallow AC, no bleb leak	Observation	BCVA: 6/60, IOP: 12 mmHg
Day 8	Second follow‐up	Persistent shallow AC, peaked pupil	First revision: Iris reposition, flap sutures, AC reformation	BCVA: 6/36, IOP: 16 mmHg
Day 19	Post first revision follow‐up	Recurrent shallow AC	Patient admitted intense sexual intercourse, second revision with sexual abstinence	BCVA: 6/36, IOP: 18 mmHg
Day 20	Post second revision follow‐up	Well‐formed AC	Continued medication	BCVA: 6/36, IOP: 14 mmHg
Day 30	Late follow‐up	Corectopia, flat bleb	Timolol maleate (0.5%) added, digital massage	BCVA: 6/12, IOP: 24 mmHg

Abbreviations: AC, Anterior chamber; BCVA, Best‐corrected visual acuity; IOP, Intraocular pressure.

We frequently focus on the surgical aspects after any glaucoma filtration procedure and ignore the patient factors associated with the postoperative shallowing of the AC. Giving patients thorough instructions, such as refraining from intense sexual activity in the early postoperative period when appropriate, may help prevent the AC from frequently shallowing after trabeculectomy due to patient‐related factors.

His last follow‐up at 3 months postoperatively showed BCVA of 6/9 and IOP of 10 mmHg (with timolol maleate). On slit lamp examination, there was a corectopia (Figure 3J) due to the iris incarcerated in the sclerostomy wound, but the bleb was moderately vascular and diffusely functioning (Figure 3K) as the iridectomy and sclerostomy were partially patent (visible with gonioscopy). The patient did not have any significant complaints other than mild photophobia due to corectopia. Later, we scheduled a trabeculectomy with MMC in LE.

## 4 Discussion

With an incidence ranging from 2% to 41%, shallow AC following trabeculectomy is one of the concerning early postoperative sequelae [1].

Patients with angle closure glaucoma are at increased risk for developing a flat AC following glaucoma filtration surgery [7], which is associated with prolonged ocular hypertension and a sudden decrease in IOP [8]. Our patient likewise had a 35 mmHg acute angle closure crisis in RE, which was not managed with maximal tolerable AGMs. Later, the patient had a trabeculectomy, and after the procedure, shallow AC occurred.

Many surgical factors, including conjunctival bleb leak, ciliochoroidal detachment, hypersecretion of aqueous humor associated with ocular inflammation, cyclodialysis cleft development, malignant glaucoma, and over‐filtration due to loose closure of the scleral flap, can cause a flat AC after trabeculectomy [2]. Compared to a limbus‐based flap, it has been noted to be more frequent after trabeculectomy using a fornix‐based flap [7]. Our patient underwent augmented trabeculectomy with a fornix‐based peritomy and triangular scleral flap with an expert glaucoma surgeon, which was uneventful.

There are many factors related to the surgical procedures, as mentioned earlier, but no literature has reported patient factors that may contribute to early postoperative shallow AC. Here are some proposed patient factors like unintentional eye rubbing, lifting heavy weights, violent coughing, sneezing, chronic constipation, or even intense physical activities, including sexual intercourse, in the early postoperative period may lead to shallow AC in the early days after filtration surgery. There are some possible mechanisms, like the Valsalva maneuver, which can significantly increase the IOP and narrow the AC angles [9], and scleral flap wound dehiscence [10], through which aqueous can easily escape immediately. So even after uneventful filtration surgery, we may face shallow AC in early postoperative periods. In this case, we encountered a shallow AC from the 1st POD, and even after the 1st revision surgery to reform the AC due to an unrecognized patient factor, probably due to intense sexual activities, in his early postoperative days.

If an optimal AC depth is not restored, peripheral anterior synechiae will obliterate the AC angle or a bleb will not form, which will result in the loss of IOP control. Furthermore, malignant glaucoma following aqueous misdirection may result from cilio‐lenticular obstruction [7]. Although conservative care is feasible in most of these situations, it is important to identify the etiology as soon as possible and to consider prompt management, including surgery, if necessary, in order to minimize consequences [7].

For the treatment of shallow AC following trabeculectomy, a number of effective techniques have been proposed, including mydriatics, intracameral injection of BSS, viscoelastic devices (OVD), and gases such as air, sulfur hexafluoride (SF6), and perfluoropropane (C3F8) [7]. In our case, we found that a combination of BSS and air in the AC for an optimal duration of time helped manage the condition. The air bubble stays in the AC until it is gradually replaced by water, which deepens the AC and prevents the lenticulo‐corneal touch.

Fibrous tissue scarring of the filtering bleb considerably lowers the success rate of glaucoma filtration surgery, and this is more common in younger patients [2]. Even though we augmented the trabeculectomy with MMC to reduce the risk of bleb failure, we kept this young patient under close follow‐up to deal with it in the long run, as we had to do revision surgeries twice in our case.

This case report describes a single patient with a recurrent shallow AC following trabeculectomy, where postoperative sexual activity was identified as a potential contributing factor. However, the association remains observational and anecdotal, as no objective measurement was available to confirm causality. Other subtle or subclinical factors, such as transient overfiltration or unrecognized structural changes, cannot be entirely ruled out. Therefore, while the hypothesis is plausible and clinically relevant, further cases and studies are required to establish a stronger correlation.

## Author Contributions

**Md. Iftekher Iqbal:** conceptualization, data curation, formal analysis, investigation, methodology, resources, validation, visualization, writing – original draft, writing – review and editing. **Fariah Osman:** data curation, formal analysis, writing – original draft.

## Ethics Statement

The Institutional Review Board of Bangladesh Eye Hospital and Institute waives the requirement for case reports.

## Consent

Written informed consent was obtained from the patient to publish this report in accordance with the journal's patient consent policy.

## Conflicts of Interest

The authors declare no conflicts of interest.

## Data Availability

No additional data were generated other than the ones mentioned in this study.
